# Dental Microwear Evidence of Holocene Dietary Variability in the Argentinian Pampas and Paraná Delta

**DOI:** 10.1002/ajpa.70330

**Published:** 2026-08-31

**Authors:** Maria Rita Guedes Carvalho, Pablo G. Messineo, María Agustina Ramos van Raap, Gustavo Flensborg, Gustavo Martínez, Nahuel A. Scheifler, Mariela E. Gonzalez, María Clara Álvarez, Cristian A. Kaufmann, Mariano Bonomo, Clara Scabuzzo, Gustavo G. Politis, Sireen El Zaatari, Katerina Harvati

**Affiliations:** ^1^ Paleoanthropology, Institute for Archaeological Sciences and Senckenberg Centre for Human Evolution and Palaeoenvironment, Department of Geosciences Eberhard Karls University of Tübingen Tübingen Germany; ^2^ Instituto de Investigaciones Arqueológicas y Paleontológicas del Cuaternario Pampeano (INCUAPA), UNCPBA‐CONICET, Olavarría, Buenos Aires, Argentina Universidad Nacional del Centro de la Provincia de Buenos Aires (UNCPBA), Facultad de Ciencias Sociales Olavarría Buenos Aires Argentina; ^3^ DFG Center for Advanced Studies “Words, Bones, Genes, Tools: Tracking Linguistic, Cultural and Biological Trajectories of the Human Past” Eberhard Karls University of Tübingen Tübingen Germany; ^4^ CONICET. División Arqueología, Facultad de Ciencias Naturales y Museo Universidad Nacional de La Plata Buenos Aires Argentina; ^5^ Centro de Investigación Científica y de Transferencia Tecnológica a la Producción (CONICET) Diamante Argentina; ^6^ Department of Anthropology of the Americas University of Bonn Bonn Germany; ^7^ EXC 3101 “HUMAN ORIGINS ‐ Excellence Cluster for Integrative Human Origins Studies” University of Tübingen Tübingen Germany

**Keywords:** Argentinean Pampas I Paraná Delta, dental microwear, hunter‐gatherers, middle and late Holocene

## Abstract

**Objectives:**

This study explores the subsistence strategies of Middle‐to‐Late Holocene human populations in the Argentinean Pampas and Paraná River Delta by analyzing dental microwear patterns.

**Materials and Methods:**

We examined occlusal molar microwear textures of individuals from the Middle and Late Holocene recovered from 12 archaeological sites across three ecologically distinct areas: the Upper Delta of the Paraná River, Central Pampas, and the lower Colorado River Valley.

**Results:**

Our results reveal both expected and unexpected trends. Individuals from the resource‐rich Upper Delta of the Paraná River exhibited the predicted high dietary diversity (indicated by elevated mean heterogeneity (*Hasfc81*) values) but also showed surprisingly high mean complexity values (*Asfc*)—exceeding even those from the two semi‐arid areas (Central Pampas and lower Colorado River Valley), where environmental abrasives would have been expected to have resulted in greater microwear texture complexity.

**Discussion:**

Within the Paraná Delta, significant intra‐site variation at Los Tres Cerros 1 suggests differential food access, possibly tied to social stratification in the Goya‐Malabrigo archaeological entity. In the Central Pampas, Middle Holocene individuals from the Arroyo Seco 2 archaeological site displayed greater dietary heterogeneity than contemporaneous and Late Holocene sites, contradicting expectations of progressive diversification during this period. These findings underscore that prehistoric subsistence strategies emerged from complex interactions between ecological constraints and cultural practices, including food processing and social organization.

## Research Highlights

- Dental microwear analysis shows unexpectedly high dental complexity in Paraná Delta populations, exceeding even semi‐arid Pampas groups.
- Intra‐site variation at Los Tres Cerros 1 suggests unequal food access, possibly linked to social stratification in Goya‐Malabrigo societies.
- Central Pampas results challenge assumptions of uniform Middle Holocene dietary restriction followed by Late Holocene diversification.

## 1 Introduction

**FIGURE 1:**
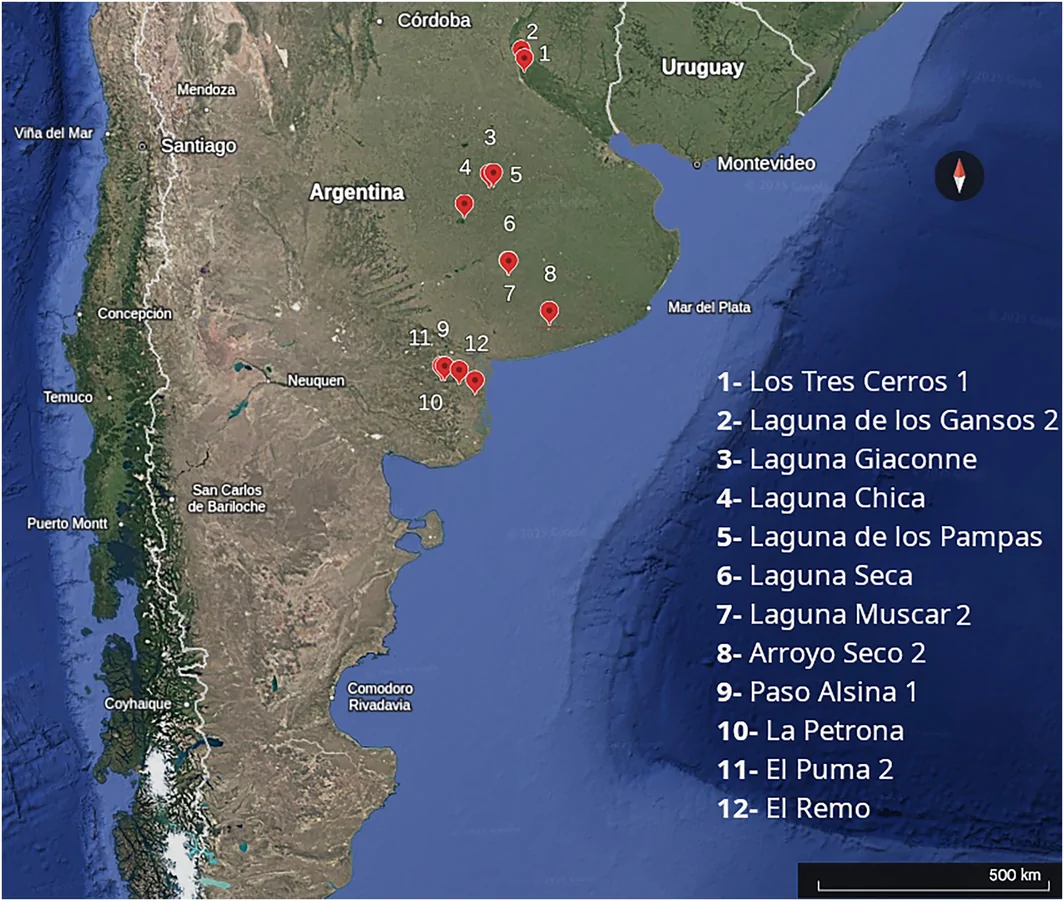
Location of the archaeological sites.

The investigation of dietary adaptations in ancient populations provides crucial insights into human–environment interactions. As access to food resources fundamentally shapes human dispersals, expansions, and settlement patterns, subsistence strategies would then reflect adaptive responses to local ecological conditions (Cohen 2009). This study explores these dynamics among Holocene hunter‐gatherers and small‐scale horticulturalists occupying three adjacent areas in east‐central Argentina, including central Pampas, the eastern Pampa‐Patagonia transition (lower Colorado River Valley), and the Upper Paraná River Delta (Figure 1). Focusing on 12 archaeological sites, we examine communities that, while mainly practicing hunter‐gatherer subsistence, are suggested to have developed diverse food procurement strategies in response to their particular environmental pressures. Evidences for such dietary differences manifest in the bio‐archaeological record as variations in faunal assemblages, archaeobotanical remains, human isotopic values, and lithic technologies that correlate with both temporal (Middle to Late Holocene) and spatial distribution across ecological gradients—from the resource‐rich Paraná Delta wetlands to the more arid lower Colorado River Valley and Central Pampas (Álvarez 2018; Flensborg et al. 2020, 2023; Scheifler 2019; Scheifler et al. 2024; Politis et al. 2009; Martínez et al. 2016; Bonomo et al. 2019). Table 1 provides the complete number of individuals included in the study, separated by period, area, and site.

**TABLE 1:** Sample summary.

Area	Site	Dates (approx.)	*N*
Middle holocene			34
Central Pampas	Arroyo Seco 2	7692–6671 BP	15
Central Pampas	Laguna de los Pampas	8413–6310 BP	14
Central Pampas	Laguna Chica	7743–6536 BP	5
Central Pampas (total)			34
Late Holocene			29
Central Pampas	Laguna Seca	1000 BP	1
Central Pampas	Laguna Muscar 2	1000 BP	2
Central Pampas	Laguna Giaccone	321–277 BP	1
Central Pampas (total)			4
Paraná Delta	Laguna de Los Gansos 2	570 ± 43 BP	2
Paraná Delta	Los Tres Cerros 1	849–650 BP	16
Paraná Delta (total)			18
Colorado River	Paso Alsina 1	436–504 BP	11
Colorado River	El Puma 2	1548 BP	1
Colorado River	La Petrona	800–250 BP	1
Colorado River	El Remo	250 BP	1
Colorado River (total)			14

To further explore these documented differences in dietary patterns, we employed dental microwear texture analysis, a repeatable and quantitative method that, when applied to the occlusal surfaces of the human molars, allows for the reconstruction of the physical characteristics of the foods consumed a short period before an individual's death (Teaford and Oyen 1989). This technique has proven particularly valuable for detecting subtle variations in human diets that might otherwise remain invisible in the archaeological record (e.g., Teaford et al. 2017; Teaford and Oyen 1989; Scott et al. 2006; Ungar and Spencer 1999). When combined with other archaeological evidence linked with food consumption, this approach provides a more comprehensive understanding of subsistence strategies. It allows us to examine both broad patterns of resource use and finer‐scale population/individual dietary behaviors.

Archaeological findings indicate that human populations first entered northeast Argentina around 12,200 BP and settled the Pampean region sometime at the terminal Pleistocene (12,170–11,180 BP) (Politis et al. 2016; Politis and Borrero 2024). During the Late Pleistocene (ca. 12,000–10,000 BP), the region was characterized by predominantly cold and semi‐arid to arid climates (De Francesco et al. 2022; Tonello and Prieto 2010), with the notable exception of the Paraná Delta, which even though passes through the Pampas biome, has formed a biodiverse hotspot within the La Plata Basin featuring a complex network of islands, channels, and lagoons that created more humid local conditions (Kaufmann et al. 2004; Cavallotto et al. 2002; López 2001). Archaeological evidence indicates that the earliest Pampean settlers had broad‐spectrum diets including megafauna and large mammals as well as smaller mammals and birds (Martínez et al. 2016; Politis et al. 2019; Politis and Borrero 2024). Throughout the Early Holocene the inhabitants of the region maintained a generalist subsistence strategy, marked by high residential mobility, logistical planning, and elaborate lithic technology (G. A. Martínez 2006; Barros and Politis 2025; Politis and Borrero 2024). While human presence during the Late Pleistocene and Early Holocene is confirmed at multiple sites (e.g., Arroyo Seco 2, Paso Otero 5, Campo Laborde; Flegenheimer et al. 2015; Politis et al. 2016, 2019), the current study focuses on the Middle and Late Holocene periods, from which well‐dated human dental remains that are better preserved and thus better suited for this study are available.

The Middle Holocene (6500–3500 years BP) marked a period of significant transformation for Pampean hunter‐gatherer societies. As the availability of high‐ranking prey declined, these societies experienced demographic fragmentation, and expanded into previously unexplored environments (Bonomo, Leon, et al. 2013). This dispersal aligns chronologically with initial dietary diversification—including the adoption of new resources—as is reflected by the technological innovations (Álvarez 2014; Barros et al. 2014). Archaeological evidence from this period reflects heightened technological complexity, with assemblages featuring diverse toolkits: large and medium‐sized stemless triangular projectile points, scraping tools (*side and end* scrapers), denticulates, notches, and boné technology (Álvarez 2014; Messineo, Barros, et al. 2019; Barros and Politis 2025). The isotopic values of Early‐Middle Holocene humans from Central Pampean sites (including Arroyo Seco 2, Laguna Chica and Laguna de los Pampas) indicates that individuals obtained protein mainly from terrestrial herbivores, which were consumers of C3 plants (Scheifler et al. 2024; Politis et al. 2009). In contrast, isotopes of human bones from sites located at the Eastern Pampa–Patagonia transition, such as Paso Alsina 1, El Puma 2, El Remo, and La Petrona have demonstrated that they developed specialized coastal adaptations, exploiting marine resources (Bonomo, Scabuzzo, and Leon 2013; Flensborg et al. 2020).

The Late Holocene in the Pampas (post‐3500 BP) is characterized by significant demographic growth. This pattern is evidenced throughout the region by increased archaeological visibility, including a greater number of larger sites and more frequent reoccupation—all interpreted as indicators of population growth (Politis and Madrid 2001; Martínez et al. 2015; Mazzanti 2006). This expansion is reflected not only in quantitative terms but also in qualitative changes, particularly through the diversification of site types and functions. The archaeological record now includes residential base camps, specialized activity areas, formal burial areas, and ceremonial sites, among others, demonstrating more complex patterns of land use and social organization of hunter‐gatherers (Martínez et al. 2016; Martínez et al. 2017; Messineo et al. 2023).

This period was also marked by significant technological developments that seem to have reshaped subsistence systems. Archaeologists have identified several key innovations, including the emergence of ceramic technology, the introduction of bow‐and‐arrow systems, and the expanded use of grinding implements (Politis and Borrero 2024). These technological advances collectively enabled more intensive exploitation of both faunal and floral resources, potentially leading to greater dietary breadth and more efficient resource processing (Politis and Madrid 2001, Martínez and Gutiérrez 2004, Bonomo 2005).

Our study aims to shed light on changes in subsistence patterns through time and space within the Pampas through the inclusion of individuals from archaeological sites from the three areas dating to the Middle and Late Holocene. The individuals included in this study are distributed across a wide temporal and geographic spectrum, implying exposure to significant ecological variation. This variability would have dictated food resource availability, potentially generating distinct dietary patterns. Consequently, our analysis focuses on how these spatial and temporal variations in subsistence strategies reflect human adaptive responses to ecological diversity in the region.

## 2 Material and Methods

### 2.1 Sample

To contextualize the study sample, we outline the ecological conditions and key archaeological features of the three areas from which the individuals originated: the Upper Delta of the Paraná River, the Central Pampas, and the lower Colorado River Valley.

#### 2.1.1 Upper Delta of the Paraná River

According to palaeoenvironmental reconstructions (Cavallotto et al. 2005), this territory only became available for human settlements after 6000 BP, when it was less subject to tidal influences (Castiñeira Latorre et al. 2013, 2017). The Paraná Delta is ecologically distinct from its surroundings due to its position within one of South America's three major river basins—alongside the Amazon and Orinoco—featuring a mosaic landscape of fluvial networks, wetlands, and terrestrial ecosystems. This dynamic hydrology ensures continuous renewal of aquatic resources, while seasonal flooding enriches soils, sustaining highly productive grasslands and diverse flora and fauna (Bonomo et al. 2019). The modern delta hosts rich forests, large mammals such as marsh deer, coypu, and capybara, hundreds of bird species, reptiles, mollusks, and around 300 species of fish, making it one of the most productive fishing regions on the continent (López 2001). Archaeologically, the studied sites in this area are linked to the Goya‐Malabrigo archaeological entity, characterized by distinctive pottery and associated in historical times with the Chaná‐Timbú ethnic groups, who occupied this area from around 2000 BP until the European conquest. Building mounds is typical of this cultural entity, probably as a means for the protection of settlements, crops, and graves from flooding (Politis and Bonomo 2012, 2023). The economy of these groups was based on a mix of resources from fishing, hunting of aquatic and terrestrial mammals, complemented by the gathering of wild plants and products from small‐scale horticulture, such as maize, squash, and beans (Bonomo et al. 2017, 2019).

Our sample comprised 18 individuals from 2 archaeological sites in this area: 2 of them recovered from Laguna de Los Gansos 2 site (570 ± 43 BP, Bonomo et al. 2016) and 16 from Los Tres Cerros 1 site (849 ± 45 BP to 650 ± 70 BP; Politis et al. 2011; Scabuzzo et al. 2015).

### 2.2 Central Pampas

Under this label are the Interserrana and the Central Pampean Dunefield areas. They are considered as a single study unit in this study due to their geographic proximity and demonstrated similarities in archaeofaunal remains. In the Middle Holocene, the subsistence patterns of human populations inhabiting this area shifted from generalized foraging to marked dietary specialization on artiodactyls—particularly guanaco (Alvarez et al. 2013; Álvarez 2014). In the Late Holocene, when archaeological sites became bigger, more numerous, and a process of technological complexification took place, archaeological evidence suggests a growing reliance on plant resources. This trend is most clearly evidenced by two key technological innovations: the introduction of ceramics and a significant expansion in grinding technologies (Messineo et al. 2023; Politis and Borrero 2024). Isotopic information also indicates an increase in human dietary breadth in the Central Pampean Dunefiels during the Late Holocene, with a greater relevance of small prey of high trophic levels (armadillos) and vegetables (Scheifler et al. 2024).

The study sample comprised 38 individuals from 6 archaeological sites across this area. These include: 15 individuals from Arroyo Seco 2 (7692–6671 BP), 14 from Laguna de los Pampas (8413–6310 BP), 5 from Laguna Chica (7743–6536 BP), 2 from Laguna Muscar 2 (≈1000 BP), and 1 individual each from Laguna Seca 1 (≈1000 BP) and Laguna Giaccone (321–277 BP).

#### 2.2.1 Lower Colorado River Valley

In This area, located in the Eastern Pampa–Patagonia transition, human occupations only took place after 6000 BP (Martínez et al. 2024). Zooarchaeological evidence shows that the inhabitants diet consisted of terrestrial foods such as guanaco, large rodents (coypu), birds, armadillos, and freshwater fish between 6000 and 3000 BP. During the initial Late Holocene (between 3000 and 1000 BP), few animal species were exploited by hunter‐gatherer groups, predominantly guanaco, Pampean deer, and large‐sized birds, however, a process of intensification is registered in the economy of the area throughout the final Late Holocene (between 1000 and 250 BP). This was also a moment of populational growth, when subsistence strategies became more diverse, with a remarkable diversification in the zooarchaeological record (Martínez et al. 2017). Smaller birds, rodents, armadillos, marine and freshwater fishes were included in the diet. Additionally, isotopic analyses of human bones from sites such as Paso Alsina 1, El Puma 2, El Remo, and La Petrona have demonstrated that they developed specialized coastal adaptations, exploiting marine resources (Flensborg et al. 2020). Besides that, substantial changes in the oral health of these populations, which also showed a significant increase in carbohydrates consumption (G. A. Flensborg 2013).

Our study included 14 individuals from this area: 11 from the Paso Alsina 1 archaeological site (436–504 BP), one from El Puma 2 (1548 BP), one from La Petrona (800–250 BP), and one from El Remo (250 BP).

### 2.3 Methods

After receiving approval from curators to access and study the original material, specimen preparation proceeded as follows: at the host institutions (see Acknowledgements session for repository details), silicone molds of dental crowns of 70 individuals were made using Coltene President Regular Body material. These molds were then used to produce positive high‐resolution epoxy replicas (Epo‐Tek 301, Epoxy Technologies) at the Paleoanthropology laboratory, University of Tuebingen.

The high‐resolution replicas of the molar teeth were used to obtain microwear texture data. The occlusal surfaces were scanned using a Sensofar PLu Neox white‐light confocal imaging profiler (Sensofar, Barcelona, Spain). Surfaces were first scanned at low magnification (10×) to determine their suitability for further study. During this procedure, 20 individuals were excluded for lacking well‐preserved surfaces due to heavy levels of macrowear or post‐mortem destruction (due to taphonomic effects or excavation/post‐excavation damage). Scans representing the remaining 50 individuals with well‐preserved pre‐mortem microwear features were taken from the parts of the occlusal surface that coincide with the second stage of the masticatory process, where food particles are grinded against the tooth (Meier and Schneck 1982). Following microwear conventions, scans were taken at 100× magnification, covering an area of 242 × 181 μm, with a lateral sampling interval of 0.17 μm and a vertical step of 0.2 μm (with the reported vertical resolution of the instrument being < 0.001 μm).

For the remaining 50 individuals, we obtained 178 scans in total. Out of these, a single optimal scan of one molar was selected to represent each individual, prioritizing second molars, when possible, but including first and third molars when preservation quality warranted. Microwear data from different molar types has been shown to be comparable within single individuals and thus its combination to increase sample size is possible (e.g., El‐Zaatari 2010). All selected scans were processed using MountainsMap software (Digital Surf 2023) using several filters and settings (see Supporting Information S1).

We then generated three standard microwear texture parameters from each surface scan: complexity (*Asfc*), anisotropy (*epLsar*), and heterogeneity (*HAsfc81*) (Scott et al. 2006).

### 2.4 Complexity (*Asfc*)

Complexity (*Asfc*) is a quantification of the tooth surface roughness across different scales of observations, and serves as a proxy for dietary abrasiveness (Scott et al. 2006). Higher *Asfc* values indicate greater surface coarseness caused by masticated abrasive particles, whether food (e.g., phytoliths, grit from unprocessed plants) or non‐food (e.g., environmental dust adhering to food). This metric effectively distinguishes between diets rich in hard, brittle foods, leading to high complexity and those that are not rich in such foods (e.g., Scott et al. 2006) or those where such foods are processed in a way that reduces their mechanical abrasiveness (e.g., El‐Zaatari 2010). Compared to humans with other forms of subsistence basis (e.g., agriculturalists), hunter‐gatherers commonly show high complexity levels because processing techniques that substantially soften foods (e.g., cooking, fine grinding) are less commonly used by them, even if other forms of processing are sometimes employed. Exceptions occur when hunter‐gatherers consume naturally soft or non‐abrasive foods, or when agriculturalists (e.g., the Kish in Iraq, ~5000 BP) regularly eat hard foods, or also when environmental abrasives interfere with the dietary signal (El Zaatari et al. 2016; Schmidt et al. 2019). Considering the environmental differences between the three areas, it is expected that samples from the Colorado River Valley show the highest complexity values due to the aridity of the ecotone where they are located, followed by the individuals from the Central Pampas, where dunefields could also mobilize eolian abrasives. Additionally, because evidence of plant consumption is more frequent at Late Holocene sites, the associated higher intake of phytoliths is expected to increase complexity levels relative to Middle Holocene sites.

### 2.5 Anisotropy ( *epLsar* )

Measures the directionality of surface striations, which form in response to repetitive chewing of tough or elastic foods. Although anisotropy often inversely correlates with complexity (since tough foods typically exhibit lower hardness), this relationship is not always maintained. For example, diets combining both abrasive and tough components (e.g., fibrous plants with adherent grit) may produce similar intermediate values for both parameters, while uniformly soft diets result in both low anisotropy and low complexity (El‐Zaatari 2010; Schmidt 2010). Schmidt et al. (2019) has found significant differences in anisotropy levels when comparing foragers with pastoralists and farmers, with foragers showing lower levels of anisotropy. Here, where all compared populations are foragers (or mainly foragers), levels of anisotropy are expected to be consistent across sites and areas.

### 2.6 Heterogeneity ( *HAsfc81* )

Reflects variability in surface complexity across the different areas of a single scan, capturing dietary diversity (Scott et al. 2006). Consuming foods with divergent mechanical properties—which produce varying levels of complexity across the occlusal surface—generates greater textural variation across this surface. Consequently, higher heterogeneity values signal eclectic dietary habits, whereas lower values indicate monotonous consumption patterns (El Zaatari et al. 2016). In this study, individuals from the Parana Delta are expected to show the highest levels of heterogeneity as the environment they lived in is characterized by the abundance of aquatic and terrestrial resources. Within the Central Pampean area, Late Holocene sites are expected to show greater heterogeneity than Middle Holocene sites, reflecting evidence of dietary diversification during the later period.

Statistical analyses were used to assess the differences in these parameters across time and space. All data underwent square‐root transformation prior to statistical analysis to mitigate potential violations of parametric test assumptions. Differences in the three variables across geographical space were assessed using a nested multivariate analysis of variance (MANOVA) with geographical area and archaeological site used as categorical variables. For the Central Pampas, another nested MANOVA model was used to compare values for the three variables through time (Middle and Late Holocene) and archaeological site. When significant differences were detected using a significance threshold of α = 0.05, analyses of variance (ANOVA) were conducted for each variable, followed by post hoc pairwise comparisons (Tukey's HSD and Fisher's LSD) to identify the specific source(s) of the significance. All statistical analyses were performed in R (R Core Team 2025).

## 3 Results

The values of the three parameters for every tooth analyzed are presented in Table 2.

**TABLE 2:** Microwear texture parameters for the individuals analyzed in this study.

Site	Area	Holocene period	Specimen ID	Tooth^a^	epLsar	Asfc	HAsfc81
Arroyo Seco2	Central Pampas	Middle	12950	LRM3	0.003408	0.4681	0.7917
Arroyo Seco2	Central Pampas	Middle	SK24	ULM2	0.00169	0.5340	0.5081
Arroyo Seco2	Central Pampas	Middle	SK5	LRM1	0.002757	0.5716	0.7254
Arroyo Seco2	Central Pampas	Middle	FCSAS213969	ULM1	0.0001684	0.6085	0.6007
Arroyo Seco2	Central Pampas	Middle	SK23	LLM1	0.002144	0.6574	0.6738
Arroyo Seco2	Central Pampas	Middle	SK36	LRM1	0.0009758	0.6841	1.085
Arroyo Seco2	Central Pampas	Middle	SK6	ULM1	0.002061	0.8636	0.8217
Arroyo Seco2	Central Pampas	Middle	SK8	ULM1	0.002736	0.8988	0.9659
Arroyo Seco2	Central Pampas	Middle	SK42	LLM2	0.002564	0.9467	0.5830
Arroyo Seco2	Central Pampas	Middle	SK4	LLM2	0.001536	1.296	0.6227
Arroyo Seco2	Central Pampas	Middle	SK7	ULM3	0.002181	1.488	0.8605
Laguna Chica	Central Pampas	Middle	Burial 2	LRM2	0.002394	0.5183	0.4338
Laguna Chica	Central Pampas	Middle	Burial 1	ULM2	0.001395	0.6677	0.6354
Laguna Chica	Central Pampas	Middle	Burial 2	URM1	0.001786	1.565	0.5430
Laguna de Los Pampas	Central Pampas	Middle	LLLP.S1.3 and LLLP.S1.4	URM3	0.001295	0.2828	0.5635
Laguna de Los Pampas	Central Pampas	Middle	LLLP.S2.1052	URM2	0.002691	0.5355	0.7075
Laguna de Los Pampas	Central Pampas	Middle	LLLP.S1.20	LRM2	0.001995	0.6875	0.4578
Laguna de Los Pampas	Central Pampas	Middle	L.LLP.S2.E6	LRM1	0.002127	0.9512	0.5817
Laguna de Los Pampas	Central Pampas	Middle	L.LLP.S2.E8	URM1	0.001566	1.288	0.6437
Laguna de Los Pampas	Central Pampas	Middle	LLL.S1.19	LLM2	0.001709	1.318	0.4026
Laguna de Los Pampas	Central Pampas	Middle	LLLP.S1.5134	LLM2	0.00198	1.328	0.6121
Laguna de Los Pampas	Central Pampas	Middle	L.LLP.S2.E7	LRM1	0.001607	1.466	0.6626
Laguna de Los Pampas	Central Pampas	Middle	LLLP.S2.1051	LLM2	0.001614	1.598	0.5688
Laguna Giaconne	Central Pampas	Late	L.LG.715	LRM1	0.0004867	1.387	0.4246
Laguna Muscar 2	Central Pampas	Late	FCS.LM1.37	URM1	0.00273	0.8962	0.4755
Laguna Muscar 2	Central Pampas	Late	FCS.LM1.38	URM1	0.002548	1.427	0.5528
Laguna Seca	Central Pampas	Late	FSC.LS1	ULM1	0.004333	0.1572	0.5660
Laguna de Los Gansos 2	Paraná Delta	Late	LDLG2‐1	URM1	0.0008119	1.112	0.8877
Laguna de Los Gansos 2	Paraná Delta	Late	LDLG2‐2	ULM2	0.002337	1.353	0.6768
Los Tres Cerros 1	Paraná Delta	Late	LTC1‐P5	ULM1	0.001597	1.050	0.5613
Los Tres Cerros 1	Paraná Delta	Late	LTC1‐S2‐2	URM1	0.002086	1.255	0.8632
Los Tres Cerros 1	Paraná Delta	Late	LTC1‐S2‐1	LLM1	0.002642	1.357	1.013
Los Tres Cerros 1	Paraná Delta	Late	LTC1‐S9‐2	ULM2	0.004733	1.558	0.5575
Los Tres Cerros 1	Paraná Delta	Late	LTC1‐S9	LLM1	0.001204	1.598	0.6257
Los Tres Cerros 1	Paraná Delta	Late	LTC1‐P1	LLM2	0.002833	1.716	0.9133
Los Tres Cerros 1	Paraná Delta	Late	LTC1‐P5	LLM1	0.00252	2.378	0.8956
Los Tres Cerros 1	Paraná Delta	Late	LTC1‐P2	LRM3	0.001562	2.582	0.8290
Los Tres Cerros 1	Paraná Delta	Late	LTC1‐P4	ULM3	0.0021	2.616	1.244
Los Tres Cerros 1	Paraná Delta	Late	LTC1‐A‐484	URM1	0.001721	2.978	0.3982
Los Tres Cerros 1	Paraná Delta	Late	LTC1‐P8	LLM2	0.001449	4.557	1.039
Paso Alsina 1	Colorado River	Late	Burial 8K	LLM1	0.002083	0.5585	0.6275
La Petrona	Colorado River	Late	Burial 3	URM2	0.001568	0.6955	0.7630
Paso Alsina 1	Colorado River	Late	Mandibula D	LLM1	0.001528	0.9614	0.5401
Paso Alsina 1	Colorado River	Late	Burial 10A	ULM2	0.00318	1.089	0.5654
El Remo	Colorado River	Late	Burial 1	URM2	0.0003741	1.297	0.5970
Paso Alsina 1	Colorado River	Late	Burial 10B	ULM2	0.0009772	1.375	0.7704
Paso Alsina 1	Colorado River	Late	Burial 8H	LRM2	0.001265	1.423	0.7856
Paso Alsina 1	Colorado River	Late	Burial 8	URM2	0.001468	1.525	0.6547
El Puma 2	Colorado River	Late	Burial 1	URM3	0.001961	1.722	0.5382
Paso Alsina 1	Colorado River	Late	Burial 1	ULM2	0.0009644	1.961	0.9054

^a^ Teeth positions are abbreviated in the following order: U = upper, L = lower, R = right, L = left, M = molar.

For the comparisons across geographical areas, the nested MANOVA model indicates a significant difference in microwear textures of individuals when grouped by geographical area (Table 3). Subsequent ANOVA models reveal that this distinction is primarily driven by two parameters, complexity (*Asfc*) and heterogeneity (*HAsfc81*), for the comparisons by area.

**TABLE 3:** Comparisons between subregion and archaeological sites.

MANOVA (subregion and site)	Pillai	*F*	*p*
Subregion	0.54308	4.7217	< 0.001
Subregion/site	0.65838	1.5665	0.0694640

ANOVA (subregion)	df	*F*	*p*
*Asfc*	2	14.76	< 0.001
*epLsar*	2	1.629	0.209
*HAsfc81*	2	4.307	0.0204

**FIGURE 2:**
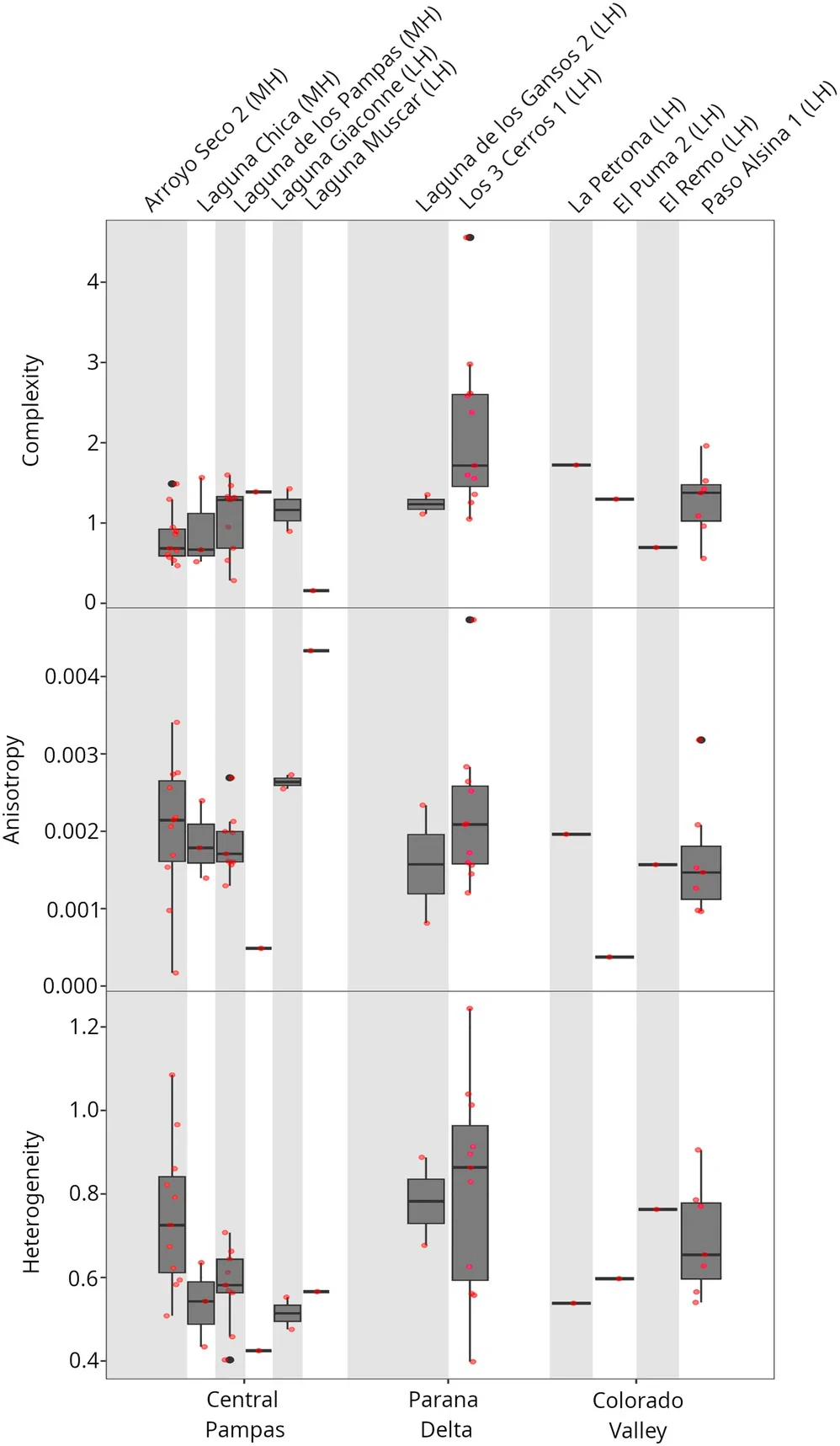
Boxplots showing the distribution of complexity (Asfc), anisotropy (epLsar), and heterogeneity (Hasfc81) for each site, grouped by geographical area (Central Pampas, Paraná Delta, and Colorado Valley).

Pairwise comparisons (Table 4) reveal significant differences in complexity (*Asfc*) between the Paraná Delta and the other two areas: while the Central Pampas and the lower Colorado River Valley do not show significant differences in complexity between them, the Paraná Delta stands out with the highest value, showing significant differences when compared to the other two. Regarding heterogeneity (*HAsfc81*), significant differences are observed only between the Central Pampas and the Paraná Delta, with the Parana Delta showing higher levels. Figure 2 illustrates the values of all three variables, categorized by area and site, and Table 5 gives the mean values for each site and region.

**TABLE 4:** Post hoc pairwise comparisons between subregions for *Asfc* and *HAsfc*81.

Pair	Fisher's LSD		Tukey's HSD	
	diff	*p*	diff	*p*
*ASFC* (by Subregion)				
Parana Delta—Central Pampas	0.4465445	< 0.001	0.4465445	< 0.001
Colorado River—Central Pampas	0.1694530	0.0679	0.1694530	0.1587
Colorado River—Parana Delta	−0.2770915	0.0101	−0.2770915	0.0268
*HAsfc81* (by Subregion)				
Parana Delta—Central Pampas	0.10041287	0.0056	0.10041287	0.0151
Colorado River—Central Pampas	0.02910826	0.4428	0.02910826	0.7202
Colorado River—Parana Delta	−0.07130461	0.1026	−0.07130461	0.2288

**TABLE 5:** Mean values per area and site.

Site/area	Asfc	epLsar	HAsfc81
Central Pampas (total)	0.9292667	0.002017700	0.6322185
Arroyo Seco 2	0.8197091	0.002020109	0.7489545
Laguna de los Pampas	1.0505556	0.001842667	0.5778111
Laguna Chica	0.9170000	0.001858333	0.5374000
Laguna Seca	0.1572000	0.004333000	0.5660000
Laguna Muscar 2	1.1616000	0.002639000	0.5141500
Laguna Giaccone	1.3870000	0.000486700	0.4246000
Paraná Delta (total)	2.0084615	0.002122762	0.8080231
Laguna de Los Gansos 2	1.2325000	0.001574450	0.7822500
Los Tres Cerros 1	2.1495455	0.002222455	0.8127091
Colorado River (total)	1.2607400	0.001536870	0.6747300
Paso Alsina 1	1.2704143	0.001637943	0.6927286
El Puma 2	1.7220000	0.001961000	0.5382000
La Petrona	0.6955000	0.001568000	0.7630000
El Remo	1.2970000	0.000374100	0.5970000

Table 6 presents the results of a comparison between Central Pampas individuals from the Middle and Late Holocene and subsequent post hoc tests (one‐way Anovas). The analysis, conducted using a nested MANOVA model, indicates no significant differences between the two time periods, but significant variations between the sites within the Middle Holocene (Arroyo Seco 2 versus Laguna de los Pampas and Laguna Chica). Table 7 presents the pairwise comparisons for heterogeneity (HAsfc81) within Middle and Late Holocene sites. Among Middle Holocene sites, Arroyo Seco 2 exhibits the highest heterogeneity, differing significantly from the other two sites. In contrast, no significant differences were detected among Late Holocene sites, though Laguna Seca recorded the highest value while Laguna Giaconne recorded the lowest.

**TABLE 6:** Comparisons between time periods and archaeological sites within the Central Pampas.

MANOVA (time and site)	Pillai	*F*	*p*
Time	0.21270	1.6210	0.21962
Time/site	0.93925	2.2789	0.01841

ANOVA (time/site)	df	*F*	*p*
*Asfc*	5	1.898	0.138
*epLsar*	5	2.537	0.0602
*HAsfc81*	5	3.028	0.0327

**TABLE 7:** Comparisons between HAsfc81 values of archaeological sites in the Central Pampas.

Pair	Fisher's LSD		Tukey's HSD	
	diff	*p*	diff	*p*
HA*sfc*81‐ Middle Holocene				
Laguna Chica ‐ Arroyo Seco 2	0.129246665	0.0270	0.129246665	0.2086
Laguna de Los Pampas ‐ Arroyo Seco 2	0.102518459	0.0124	0.102518459	0.1098
Laguna de Los Pampas ‐ Laguna Chica	0.026728206	0.6358	0.026728206	0.9964
HA*sfc*81‐ Late Holocene				
Laguna Muscar 2 ‐ Laguna Giaconne	0.064921764	0.5320	0.064921764	0.9869
Laguna Seca ‐ Laguna Giaconne	0.100716333	0.4029	0.100716333	0.9534
Laguna Seca ‐ Laguna Muscar 2	0.035794569	0.7296	0.035794569	0.9992

## 4 Discussion

The significant environmental variability across the three distinct geographic areas included in this study likely drove distinct subsistence strategies and dietary adaptations among their human populations. Despite the limitations in sample size (45 individuals), especially when it comes to site‐level subsamples, these differences were still observable, and they are supported by evidence from zooarchaeological studies, stable isotope data, and paleopathological research (Bonomo, Leon, et al. 2013; Bonomo, Scabuzzo, and Leon 2013; Bonomo et al. 2017; Ramos van Raap 2020; Scabuzzo et al. 2024; Scheifler 2019; Scheifler et al. 2024; G. A. Flensborg 2013). It should be noted that even though we would ideally have also aimed to assess changes in diets through finer scales of time, from the Late Pleistocene to the Late Holocene, suitable preservation of dental samples for inclusion in the study dictated a focus on the early Middle Holocene (ca. 7700–6400 cal BP) and the later part of the Late Holocene (ca. 1000–300 cal BP). Thus, our temporal analysis does not capture a gradual, continuous transition in dietary behaviors, but rather accumulated dietary shifts resulting from major environmental, demographic, and cultural changes that occurred over this time interval.

Our findings not only corroborated some well‐known dietary differences between the analyzed areas but also revealed new nuances in the human adaptive patterns. The groups inhabiting the Paraná Delta, for example, met the expectations of showing relatively high values of heterogeneity due to their access to a resource‐rich environment. This expectation was supported by ethnographic and archaeological evidence, which indicates that these groups (from the Goya‐Malabrigo tradition) practiced a broad‐spectrum subsistence strategy, combining hunting‐gathering with horticulture. They cultivated crops such as squash (*Cucurbita* spp.), maize ( *Zea mays* ), and beans (*Phaseolus* spp.), while also exploiting a wide range of fauna (Bonomo et al. 2019). Primary resources included semi‐aquatic and terrestrial mammals like coypu ( *Myocastor coypus* ), capybara ( *Hydrochoerus hydrochaeris* ), wild cavy ( *Cavia aperea* ), and cervids ( *Blastocerus dichotomus* , *Ozotoceros bezoarticus* , *Mazama* spp.), alongside intensive fishing (mainly Characiformes and Siluriformes). Secondary resources encompassed birds, reptiles, and freshwater mollusks (Politis and Bonomo 2023). This dietary breadth is further corroborated by archaeofaunal records from Los Tres Cerros 1 (the largest site included in our sample), which reveal a diverse assemblage of mammals, birds, mollusks, and abundant fish (Bastourre 2014). Also, stable isotope analyses from Goya‐Malabrigo sites (including Los Tres Cerros 1 and Laguna de los Gansos 2) indicate a strong reliance on C_3_ plants, which could derive from wild or domesticated species, as well as C_3_‐consuming terrestrial animals. Elevated δ^15^N values further suggest significant consumption of ^15^N‐enriched resources, likely fish, birds, or other fluvial taxa (Bonomo et al. 2017; Scabuzzo et al. 2024).

For the Central Pampas, on the other hand, there was an expectation of low values for heterogeneity (*HAsfc81*). Zooarchaeological evidence indicates dietary homogenization during the Middle Holocene in this area as a whole, including at one of the sites included in this study (Laguna de los Pampas, Álvarez 2017). A reduced diversity of exploited fauna—particularly a reliance on guanaco ( *Lama guanicoe* )—has been documented for this period in several sites of the Central Pampas (Alvarez et al. 2013; Martínez and Gutiérrez 2004). However, overall lower heterogeneity levels are also observed across Central Pampas sites dating to the Late Holocene (Laguna Seca, Muscar 2, and Giaccone) (Figure 2). This is somewhat surprising, given that these sites are chronologically located in a period commonly associated with dietary diversification in the broader Pampas region ‐the Late Holocene‐, as marked by technological innovations (e.g., ceramics) and increased reliance on plant resources (G. A. Martínez 2006; Mazzanti 2006; Politis and Borrero 2024; Scheifler et al. 2024). Actually, the comparison of microwear textures of individuals from the Middle and Late Holocene in the Pampas region challenges the assumptions that all populations experienced the same level of dietary homogenization during the Middle Holocene and then diversification during Late Holocene.

The wide distribution of heterogeneity values for individuals from the Paraná Delta also highlights a key dietary peculiarity in this area: substantial intra‐site variability, particularly at Los Tres Cerros 1. As illustrated in Figure 2, *HAsfc81* values at this site span a notably wide range compared to all other sites in this and the different areas, suggesting that not all individuals consumed equally diverse diets—something that has already been suggested by isotopic studies conducted in the same area (Bonomo et al. 2017). This pattern could indicate differential access to food resources within these populations, potentially tied to the social stratification seen in Goya‐Malabrigo populations (Politis and Bonomo 2023). This model of intra‐community dietary variation is further contextualized by broader regional patterns: a multivariate diet reconstruction by Scabuzzo et al. (2024) for the area suggests a baseline diet of continental resources based on C3 plants, with occasional C4 foods like maize. Crucially, their model also confirmed significant dietary differences *between individuals from different sites*, underscoring that dental complexity in the area operates at multiple scales—both within and between communities.

Although our sample does not have associated information on social status of specific individuals in Los Tres Cerros 1, ethnohistorical and archaeological evidence indicates that the Chana‐Timbu societies (associated with Goya‐Malabrigo tradition) were ranked, with some individuals enjoying preferential access to exotic and prestige goods, like copper and bronze objects (Politis and Bonomo 2012, 2023; Politis and Tissera 2023). Thus, the dietary variability we observe within a single archaeological entity could indeed signal such social differentiation. However, we also recognize that part of the observed intra‐site variation may be influenced by sample size differences. This, however, does not seem to be determinative as illustrated in our other sites which have similar sample sizes yet much lower intra site variation of heterogeneity or other parameters (e.g., Lagua de la Pampas in central Pampas, Paso Alsina 1 in Colorado River Valley).

Aside from heterogeneity, another microwear texture parameter significantly differentiating individuals across geographic areas was dental complexity, which measures the level of abrasiveness in the diet. Previous studies of overall dental macrowear have documented pronounced levels of dental wear among the lower Colorado River Valley populations, including cases of pulp exposure associated with periapical lesions and tooth loss (G. Flensborg 2016). In these studies, the macrowear patterns did not indicate paramasticatory activities, as evidenced by the lack of significant anomalous occlusal directionality (G. A. Flensborg 2013), suggesting that diet coupled with the arid, sandy environment was the primary contributing factor. Besides the persistent wind mobilizing fine abrasive particles—increasing the likelihood of sediment ingestion—the region is also rich in tough, fibrous plant resources (Powell 1985; G. A. Flensborg 2013). Given these conditions, we expected to see the highest levels of complexity (*Asfc*) in individuals from this area, followed by individuals from the Central Pampas—another area with dune fields and potential eolian abrasives.

Contrary to these predictions, however, individuals from the Paraná Delta sites exhibited the greatest average dental complexity values instead. The significant high levels of dental complexity observed in the Paraná Delta samples represent a novel finding, with the highest average value reported for individuals from the Los Tres Cerros 1 site. Previous research (Ramos van Raap 2018) has reported only moderate dental macrowear in the sites analyzed here (Los Tres Cerros 1 and Laguna de los Gansos 2), which is consistent with a mixed subsistence economy that combines foraging and horticulture and does not necessarily contradict the findings of high complexity (Schmidt 2010). Due to the lack of extreme directional drivers, mixed economies typically exhibit intermediate macrowear values when compared to agriculturalists and foragers (Deter 2009; Smith 1984). Additionally, the characteristic macrowear patterns (natural directionality and partially concave morphology) combined with the documented low prevalence of dental caries further support this mixed subsistence model (Bonomo et al. 2017, 2019; Ramos van Raap 2020).

These unexpectedly high complexity (*Asfc*) values in the sites of the Parana Delta indicate that conventional assumptions about masticatory stress in mixed economies may not fully explain the observed patterns, pointing to potentially unique environmental or cultural factors affecting dental macrowear in this area. In this regard, the high levels of heterogeneity observed in this same sample should also be considered in conjunction with these results. Potential clues may emerge from dental calculus analyses of these populations. Phytoliths have been identified (mainly from Poaceae leaves and morphotypes related to Oryzoideae), as well as starch grains (from *Zea mays* , *Phaseolus* sp., and *Neltuma* sp.); in addition to plant fibers, sponge spicules, and diatoms. Furthermore, according to the analysis of taphonomic damage on the starch grains, wild and domesticated plants were likely consumed roasted, boiled, or in the form of flour (Ramos van Raap 2020; Ramos van Rapp et al. 2025). These findings suggest two things: first, the consumption of highly abrasive plant materials; and second, a possible ingestion or use of other silica‐rich substances—whether through food preparation techniques, hygiene practices, or other cultural behaviors not typically preserved in archaeological assemblages. The combined abrasive effects of these materials may help explain the anomalous microwear patterns. However, further research on dietary habits is needed to determine the exact behavioral and environmental factors contributing to this exceptional dental complexity.

## 5 Conclusion

Dental microwear patterns across the Paraná Delta, lower Colorado River Valley, and Central Pampas demonstrate a greater degree of intra‐regional variability than expected, both between sites and within individuals of the same site. This surprisingly high variability suggests that cultural specifics have played a major role in producing the observed patterns. The case of the Paraná River Delta is particularly telling: subsistence strategies alone do not account for the observed dietary differences within its populations. Instead, likely, social organization—such as the ranked structure of the Goya‐Malabrigo/Chaná‐Timbú societies—and other cultural practices played a key role in shaping individual diets and oral health patterns. Additionally, the high levels of microwear complexity (*Asfc*) observed in this area, despite the absence of abrasive environmental elements, point to a potential significance of food preparation methods in influencing dietary texture. These findings highlight that individual dietary habits and oral health can be as sensitive to cultural variables (like food processing and cooking techniques) as they are to broader subsistence frameworks. Also, other cultural aspects, like social differentiation, can result in differential access to food, creating dietary diversity that goes beyond what environmental models would predict.

This study also challenges the notion that a uniform process of dietary restriction happened in the Pampas during the Middle Holocene, followed by a process of dietary diversification in the Late Holocene. Our results reveal a more nuanced pattern of diet variation between archaeological sites, demonstrating that these processes did not occur uniformly across all populations.

Our research ultimately demonstrates that reconstructing ancient food systems requires multidisciplinary integration of diverse data sources. Prehistoric subsistence strategies emerged from dynamic interactions between ecological possibilities, cultural traditions, and social structures. The dental evidence presented here suggests that Holocene foodways likely represented more than passive adaptations to environmental constraints, potentially reflecting active cultural choices that could produce unexpected biological signatures.

## Author Contributions

**Cristian A. Kaufmann:** writing – review and editing, data curation, resources, conceptualization. **Nahuel A. Scheifler:** writing – review and editing. **Gustavo G. Politis:** conceptualization, data curation, resources, writing – review and editing, project administration. **María Clara Álvarez:** writing – review and editing. **Gustavo Martínez:** writing – review and editing. **Maria Rita Guedes Carvalho:** writing – original draft, investigation, conceptualization, methodology, visualization, validation, writing – review and editing, software, formal analysis, data curation. **María Agustina Ramos van Raap:** data curation, writing – review and editing, conceptualization, resources. **Sireen El Zaatari:** resources, supervision, data curation, project administration, writing – review and editing, writing – original draft, funding acquisition, investigation, conceptualization, methodology, validation, visualization, software, formal analysis. **Katerina Harvati:** conceptualization, investigation, funding acquisition, writing – original draft, writing – review and editing, resources, supervision, data curation, project administration, methodology, validation, visualization, software, formal analysis. **Mariano Bonomo:** writing – review and editing. **Gustavo Flensborg:** data curation, conceptualization, writing – review and editing, resources. **Mariela E. Gonzalez:** writing – review and editing. **Clara Scabuzzo:** writing – review and editing. **Pablo G. Messineo:** investigation, methodology, visualization, writing – review and editing, data curation, resources, conceptualization, formal analysis.

## Funding

This research was supported by the Deutsche Forschungsgemeinschaft (DFG; Harvati Leibniz award 2021; DFG FOR‐2237). SEZ and KH are supported by the European Research Council (ERC‐CoG‐101,001,889 and ERC‐AdG‐101,019,659 respectively) and K.H. is also supported by the Carl Friedrich von Siemens Foundation. P.G.M., G.G.P., N.A.S. and M.E.G. were supported by National Geographic Society (grant NGS‐50543R‐18), CONICET (PIP11220210100004CO and PUE no. 0079).

## Ethics Statement

The study of human remains was carried out in accordance with national legislation (National Law No. 25.743; National Law No. 25.517) and with the (Aranda et al. 2014).

## Conflicts of Interest

The authors declare no conflicts of interest.

## Supporting information


**Data S1:** ajpa70330‐sup‐0001‐Supinfo.docx.

## Data Availability

All data generated or analyzed during this study are included in this published article. The complete dataset of dental microwear texture parameters (complexity—Asfc, anisotropy—epLsar, heterogeneity—HAsfc81) for each individual specimen is provided in Table 2 of the main text. All individual surface scans (.plu files) are available in Zenodo.
