# Supplementary material for: Dental Microwear Evidence of Holocene Dietary Variability in the Argentinian Pampas and Paraná Delta
Source: Am J Biol Anthropol. 2026 Aug 31;191(1):e70330. doi: 10.1002/ajpa.70330 (PMC13527593; doi:10.1002/ajpa.70330)
Supplement: Supplementary file 1 [file AJPA-191-e70330-s001.docx]

**Supplementary Material**

Upload the surface (.plu) on MountainsMap® software (Digital Surf, 2023), and apply:

1. **RESAMPLE:** resampling in x and y to 0.2 μm (Operators> Axes> Resample).
2. **LEVEL:** Level surface removes “spikes” rather than the corners of scans when surfaces were not scanned levelled (Operator> Adjust position> Level).
3. **REMOVE FORM:** Ensure that during thresholding, spikes are removed rather than the uppermost region of heavily curved surface (Operator> Filter> Remove Form).
4. **REMOVE OUTLIERS:** Remove surfaces with slope >80°, indicative of measurement noise spikes (Operator> Correct> Remove Outliers).
5. **THRESHOLD:** Removes noise (Operator> Correct> Threshold).
6. **STANDARD FILTER:** Removes surface roughness by using a cut-off scale of 2.5 μm ( Operator> Filter > Metrological Filter Filter type: Spline (ISO 16610-62)).
7. **RETOUCHING SURFACES:** With this tool, bubbles and other artifacts on the surface can be manually removed (Operator> Correct> Retouch).
8. **FILL IN NON-MEASURED POINTS:** (Operator> Correct > Fill NM points).
9. **EXTRACT AREA:** Extract an area of 242 x 181 μm to equalize between microscopes (Operator > Extract> Extract area).
10. **RESAMPLE:** resampling in x and y to 0.2 μm (Operators> Axes> Resample).
